# The Structural Basis for the Integrity of Adenovirus Ad3 Dodecahedron

**DOI:** 10.1371/journal.pone.0046075

**Published:** 2012-09-25

**Authors:** Ewa Szolajska, Wim P. Burmeister, Monika Zochowska, Barbara Nerlo, Igor Andreev, Guy Schoehn, Jean-Pierre Andrieu, Pascal Fender, Antonina Naskalska, Chloe Zubieta, Stephen Cusack, Jadwiga Chroboczek

**Affiliations:** 1 Institute of Biochemistry and Biophysics, Polish Academy of Sciences, Warsaw, Poland; 2 Unit for Virus Host-Cell Interactions, Université Joseph Fourier Grenoble 1/European Molecular Biology Laboratory/Centre National de Recherche Scientifique (UJF Grenoble 1/EMBL/CNRS UMI 3265), Grenoble, France; 3 Institute of Molecular Biology and Genetics, National Academy of Sciences of Ukraine, Kiev, Ukraine; 4 Institut de Biologie Structurale Jean-Pierre Ebel (IBS), Commissariat d'Energie Atomique, Grenoble, France; 5 European Synchrotron Radiation Facility, Grenoble, France; 6 Grenoble Outstation, EMBL, Grenoble, France; 7 Techniques de l'Ingénierie Médicale et de la Complexité - Informatique, Mathématiques et Applications de Grenoble, UMR 5525 CNRS/UJF Grenoble 1, La Tronche, France; 8 IBS, UJF Grenoble 1, Grenoble, France; 9 IBS UMR 5075, CNRS, Grenoble, France; Bioinformatics Institute, Singapore

## Abstract

During the viral life cycle adenoviruses produce excess capsid proteins. Human adenovirus serotype 3 (Ad3) synthesizes predominantly an excess of free pentons, the complexes of pentameric penton base and trimeric fiber proteins, which are responsible for virus penetration. In infected cells Ad3 pentons spontaneously assemble into dodecahedral virus-like nano-particles containing twelve pentons. They also form in insect cells during expression in the baculovirus system. Similarly, in the absence of fiber protein dodecahedric particles built of 12 penton base pentamers can be produced. Both kinds of dodecahedra show remarkable efficiency of intracellular penetration and can be engineered to deliver several millions of foreign cargo molecules to a single target cell. For this reason, they are of great interest as a delivery vector. In order to successfully manipulate this potential vector for drug and/or gene delivery, an understanding of the molecular basis of vector assembly and integrity is critical. Crystallographic data in conjunction with site-directed mutagenesis and biochemical analysis provide a model for the molecular determinants of dodecamer particle assembly and the requirements for stability. The 3.8 Å crystal structure of Ad3 penton base dodecamer (Dd) shows that the dodecahedric structure is stabilized by strand-swapping between neighboring penton base molecules. Such N-terminal strand-swapping does not occur for Dd of Ad2, a serotype which does not form Dd under physiological conditions. This unique stabilization of the Ad3 dodecamer is controlled by residues 59–61 located at the site of strand switching, the residues involved in putative salt bridges between pentamers and by the disordered N-terminus (residues 1–47), as confirmed by site directed mutagenesis and biochemical analysis of mutant and wild type protein. We also provide evidence that the distal N-terminal residues are externally exposed and available for attaching cargo.

## Introduction

Virus-like particles, called dodecahedron-fiber (DF), are generated during the adenovirus serotype 3 (Ad3) life cycle in infected cells [1]. These symmetrical, dodecahedral particles are composed of 12 copies of the viral penton complex, which comprises the pentameric penton base (Pb) and the attached trimeric fiber, both of which are essential for viral cell entry. It has been demonstrated that during the Ad3 infectious cycle a large excess of such dodecahedral particles is produced in comparison with Ad3 virions [1]. Ad3 fiber recognizes desmoglein 2, a component of the cellular junctions of epithelial cells. Interaction of the DF with desmoglein 2 induces cellular signaling cascades leading to an epithelial-to-mesenchymal cell transition characterized by transient opening of intercellular junctions [2]. Recent data show that DF competes with Ad3 virions for binding to the cell-surface and triggers cell remodeling [3]. In contrast, the better known serotype Ad2 uses the excess production of free trimeric fibers that interact with the cellular Ad2 receptor CAR in order to weaken tight junctions [4].

Baculovirus expression of Ad3 Pb protein alone results in formation of recombinant dodecahedra devoid of fibers, called dodecahedron-base (hereafter referred to as Dd) [5], but which nevertheless very efficiently penetrate human cells in culture – up to 300 000 Dd per cultured cell [6]. Since each particle is multivalent, with 60 copies of the Pb monomer, it can be engineered into a vector capable of delivering several millions of foreign cargo molecules into one cell [6], [7], [8], [9]. We have demonstrated that antigenic protein delivered with Dd to human dendritic cells is properly processed and presented [9], and that the use of Dd for delivery of the anticancer drug bleomycin to neoplastic cell results in a 100-fold increase in drug bioavailability [8]. However, to optimally engineer the vector for successful delivery of non-permeant and potentially labile cargoes such as proteins, peptides or small drugs under a variety of physiochemical conditions it is important to maintain the integrity and the penetration properties of the particle.

From previous work we know that the dodecameric structure of the Dd vector does not depend on disulphide bridges or cations as in other virus-like particles [10], [11], [12]. We therefore sought to identify residues of Ad3 Pb which mediate inter-Pb interactions and are thus responsible for particle integrity. We have previously described the crystal structure of a dodecahedron formed from Pb of adenovirus serotype 2 (Ad2) [13]. Interestingly, Ad2 Pb is present as free pentamers in infected cells and *in vitro* under normal buffer conditions. Only in the presence of ammonium sulphate and dioxane or high concentrations of 2-methylpentane-2,4-diol does it assemble into Dd, which closely resemble Ad3 Dd [13]. Ad3 and Ad2 Pb serotypes have a high degree of sequence identity and share the same overall fold. The Ad2 Pb monomer folds into two domains, a jellyroll-motif domain (40% of the residues) in the interior of the particle and an externally accessible insertion domain (60% of the residues). Ad2 Dd is formed by interactions between three loop regions in the jellyroll domain organized around a 2-fold axis. The contacts, however, do not form an extensive interface [13]. The Ad3 Dd structure has been previously determined using cryo-electron microscopy (cryoEM) and image analysis to a resolution of 9 Å [14]. Fitting the Ad2 Dd X-ray structure into the EM envelope of Ad3 Dd suggested that similar interactions occur between adjacent Pbs in both particles [13], [14]. However, as Ad2 Dd is formed only under particular solvent conditions and exhibits a limited inter-Pb interface, whilst Ad3 Dd is formed spontaneously during either Ad3 infection or recombinant expression [1], [15], it was conceivable that Ad3 Dd possesses additional interaction surfaces able to further stabilize the Dd particle, that were not revealed by the low resolution cryoEM structure.

The potential involvement of the Pb N-terminus in Dd integrity cannot be inferred from the published atomic structure as Ad2 Dd crystals were grown from Ad2 Pb truncated before residue 48 [13]. Similarly, in the Ad5 capsid structure described by Liu *et al.* [16] the first N-terminal 36 residues of the Pb are not visible. Based on the position of amino acid 48 as observed in the Ad2 Dd structure, it appeared that the Ad2 Pb N-terminus would be most likely located in the virion interior [13]. However, we have shown that Pb of Ad2 as well as both types of dodecahedron of Ad3 (DF and Dd) interact through the first of the two PPxY motifs in the Pb N-terminus (^18^PPVY for Ad3 Pb) with several proteins containing type 1 WW domains [17], [18], suggesting that at least in the recombinant-wild type dodecahedron (rwtDd) some of the Pb N-termini extend from the main body of the particle. Moreover, when the WW domains were expressed in fusion with a protein of interest, attachment of the WW fusion protein to the ^18^PPVY motif could be used for Dd-mediated protein translocation [6], [9], [19], demonstrating the considerable strength of this non-covalent interaction.

In view of the potential therapeutic uses of Ad3 Dd as a vector, the identification of residues controlling Dd integrity is critical for further vector manipulation. The structural, biochemical, and mutagenesis study presented here elucidates a novel strand swapping mechanism responsible for the integrity of the Ad3 Dd. This work will allow the optimization of the vector design for *in vivo* use.

## Results

### Crystallographic analysis of the Ad3 dodecahedron

**Figure 1:**
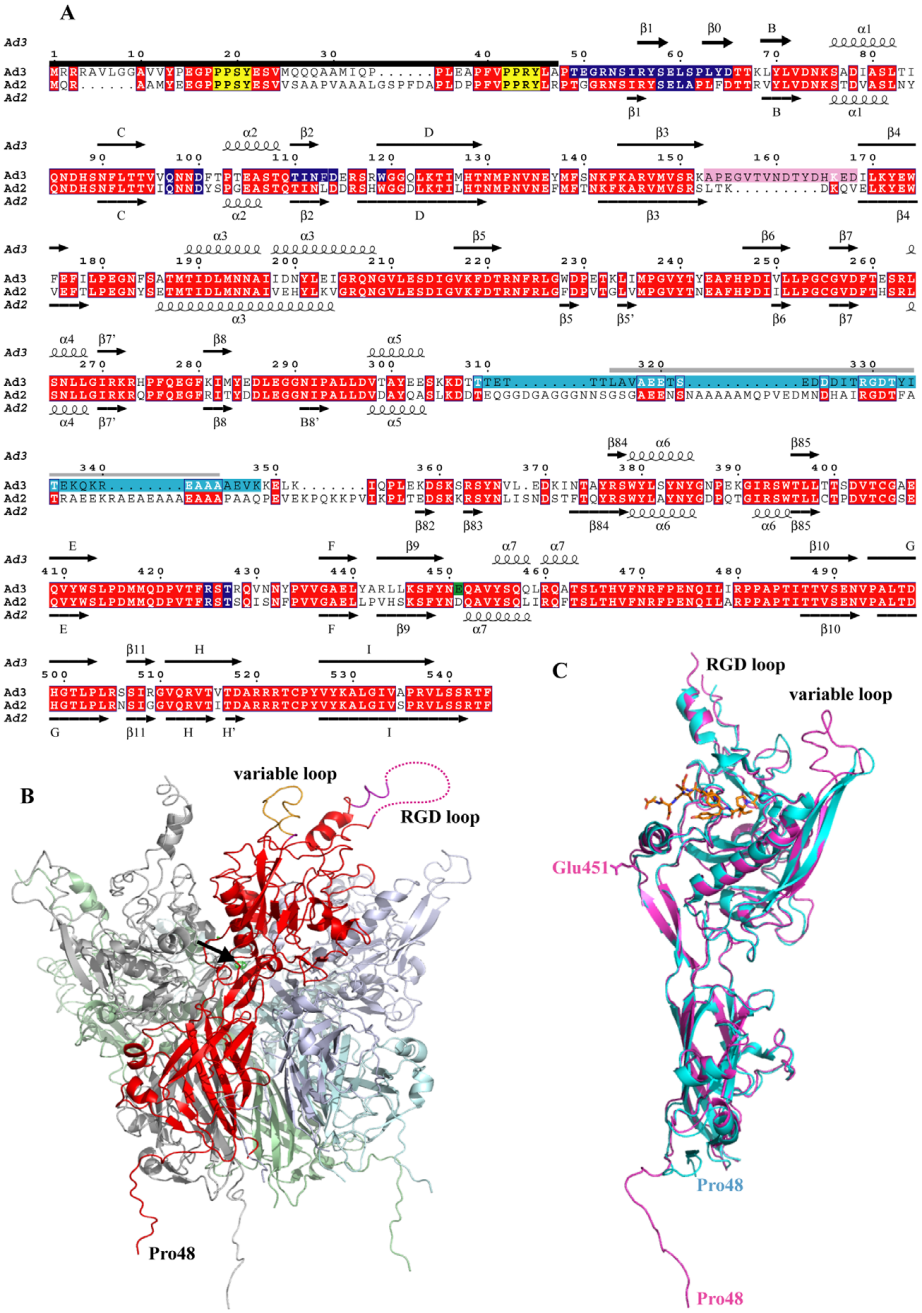
Ad3 dodecahedron structure. (**A**) The secondary structure of the Ad3 Pb in the orthorhombic crystal form is shown together with the one of Ad2 (pdb entry 1×9p [13]). The corrected sequence (GenBank acc. no ABB17799.1 [26]) of the Ad3 Pb with ^58^SELS instead of ^58^SDVS has been used. Disordered or proteolysed residues at the N-terminus which are invisible in the structure are indicated with a black bar. PPxY motifs are marked in yellow. Glu451 involved in a putative Ca^2+^ binding site is highlighted with a green background, the variable loop with a pink background, the RGD loop with a light blue background. The peptide of the RGD binding loop that is probably missing due to proteolysis, is marked with a gray bar. A dark blue background marks contact residues involved in the Dd formation. Secondary structure elements are labeled as in Zubieta *et al.* [13] whenever possible. (**B**) Cartoon representation of the overall structure of an Ad3 Pb in the cubic crystal form. One of the Pb subunits is highlighted in red. The dotted line symbolizes the part of the RGD loop, which is invisible in electron density. The black arrow points to the putative calcium ion. The ‘dangling’ N-terminal ends at the bottom are in fact involved in strand-swapping with other Pbs. (**C**) Alignment of the Ad3 Pb monomer structure (magenta) from the orthorhombic crystal form in presence of fiber peptide (orange) with the one of Ad2 (cyan).

The atomic structure of the Ad3 Dd was first determined using crystals of space group P2_1_3 in which there are 20 Pb monomers, i.e. 1/3 of the Dd, in the asymmetric unit. The structure was solved by molecular replacement using a model derived from the Ad2 Dd crystal structure and refined with an R_free_ = 0.290 at a resolution of 4.75 Å (Tables S1 and S2). The overall fold of the Ad3 Pb molecule is very similar to that of Ad2. Structural differences concern principally the variable loop (Figure 1), which is longer than in Ad2 but relatively well ordered. A second loop region containing the internalization motif RGD (hereafter called the RGD loop) is only partially visible in the Ad3 structure and rather poorly ordered. It is possible that the loop is proteolyzed in the crystal as N-terminal sequencing and mass spectrometry of Dd preparations often indicated the absence of residues 313 to 347 (data not shown).

An orthorhombic crystal form for Ad3 Dd was obtained in the presence of an N-terminal peptide derived from the Ad2/3 fiber consensus sequence. This crystal form contains one full Dd (i.e. 60 copies of the Pb monomer) in the asymmetric unit and therefore allows improvement of the electron density by 60-fold non-crystallographic averaging. Using strict 60-fold non-crystallographic symmetry, the structure has been refined to 3.8 Å resolution (R_free_ = 0.280) (Tables S1 and S2). Additional electron density, corresponding to the sequence NPVYPYDTEC, was visible in the fiber peptide-binding site of the Pb, confirming that the highly conserved FNPVYPY motif of the fiber mediates the binding as previously described [13] (Figure S1A). In addition, the conformation of residues 452 to 467 of the Ad3 Pb corresponds to that observed for Ad2 Pb when bound to the fiber peptide (pdb entry 1×9t, [13], Figure S1B).

In both Ad3 Dd crystal forms, the visible electron density for the Pb peptide chain starts at residue Pro48, while a very strong positive electron density (>10 σ and >18 σ, respectively for each crystal form) is consistently present in averaged Fo-Fc maps on the Pb 5-fold axis and coordinated by Glu451 from each subunit (Figure S1C). As Ca^2+^ was present during the crystallization, it is most likely that this density corresponds to a bound calcium ion. However, calcium does not appear to be involved in the Dd integrity as the particle is not destabilized in the presence of calcium-chelating EDTA or EGTA at 100 mM (results not shown).

### Ad3 dodecahedron assembly

**Figure 2:**
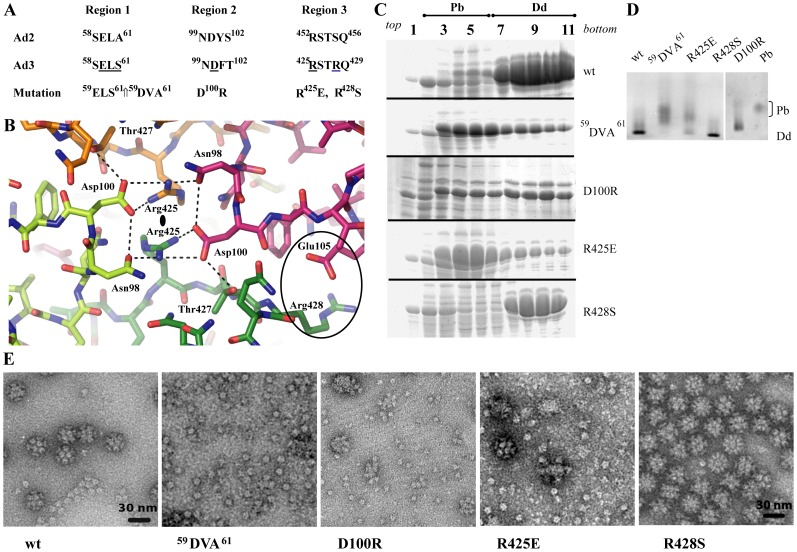
Analysis of dodecahedra formation by Ad3 Pb mutants. (**A**) Amino acid sequence of Pb protein regions responsible for Dd integrity. (**B**) Structure of regions 2 and 3 involved in Dd formation. These residues are located around the 2-fold axes of the dodecahedron. The light and dark green molecules belong to one Pb, the magenta and orange molecules to another one. Dotted lines mark potential hydrogen bonds, although they cannot be identified unambiguously due to the limited resolution. (**C**) Sucrose density gradients of rwtDd and mutants. Cell extracts obtained from expressing HF cells were fractionated and analyzed on denaturing PAGE as described in Material and Methods. (**D**) Native gel analysis of Q-Sepharose fractions eluted with 370 mM NaCl and revealed with CBB stain. Pb and wt Dd were used as internal standards. Note the shift of Dd bands due to changed net charges of Dd constructs. (**E**) Electron microscopy of wt and mutant Dd. Samples shown in **C** were analyzed after dialysis.

The Ad3 Dd is a particle with external diameter of about 290 Å and an internal cavity of 80 Å diameter. Contacts between Pb pentamers forming the Dd appear at first sight to be very limited. Relatively large (15 Å diameter) trefoil openings are located on its 3-fold axes connecting the interior to the exterior (Figure S2B). Although there is no visible electron density in the trefoil channel, it is wide enough to accommodate a disordered peptide, conceivably the presumed flexible N-terminal 47 residues of the Pb. Interactions between Pb pentamers involve the charged residues Asp100 of region 2 and Arg425 of region 3 (Figure 2AB), as previously deduced from the atomic resolution structure of the Ad2 Pb [13], [14]. In addition, these residues form a hydrogen bond network involving Asn98 and Thr427, thus stabilizing the particle.

**Figure 3:**
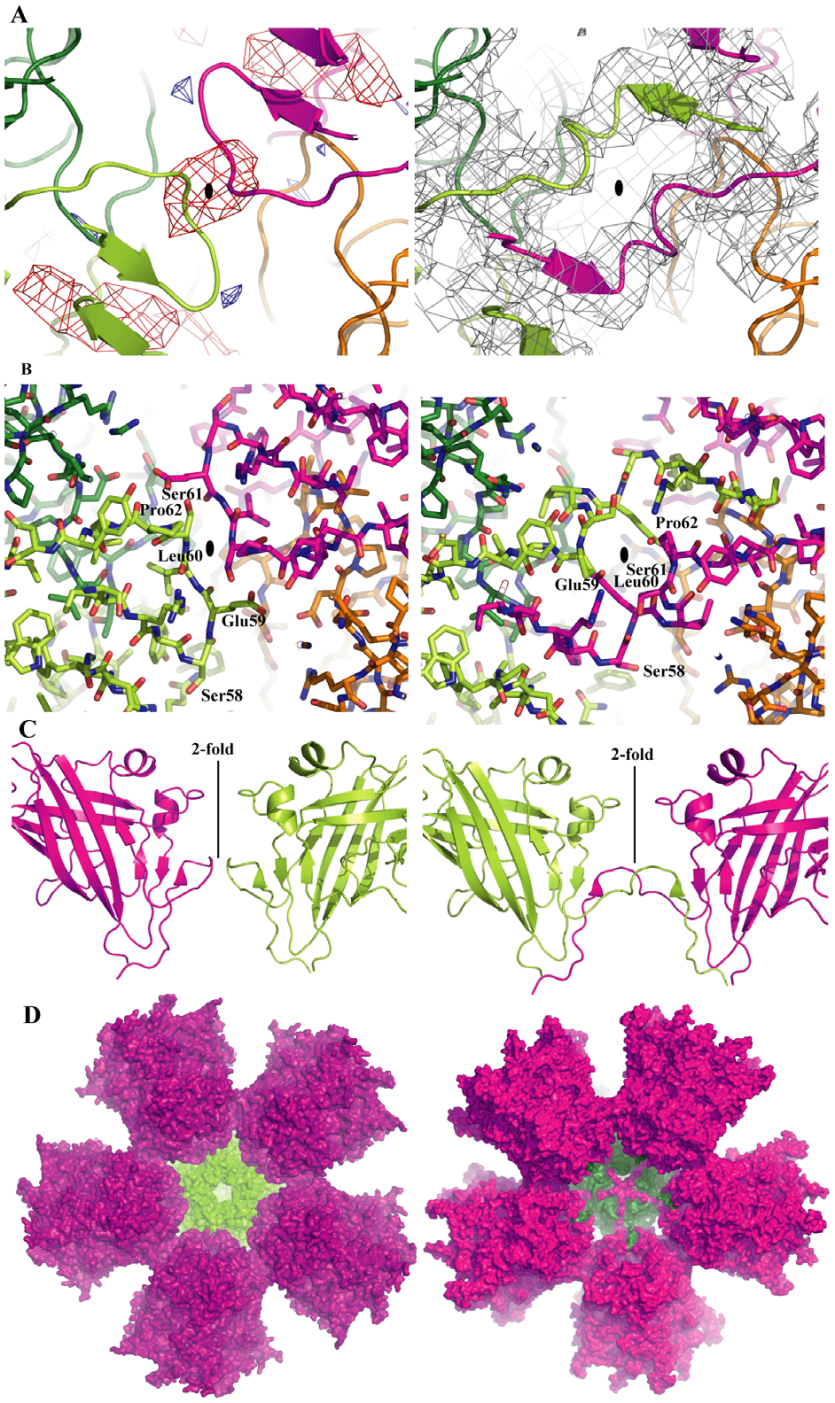
Ad3 Dd stabilization by strand-swapping. (**A**) Cartoon representation of the peptide backbone of the Dd without strand-swapping (left) and with strand-swapping (right), next to a 2-fold axis (small black oval). Residues contributed by one Pb are shown in dark green and light green, the ones contributed by the 2-fold related neighbor are shown in magenta and orange. Using model phases from the model without strand-swapping and experimental structure factor amplitudes from the cubic crystal form, 20-fold averaged Fo-Fc and 2Fo-Fc electron density has been calculated. On the left side the Fo-Fc electron density is shown (red, negative electron density; blue, positive electron density). The negative electron density indicates a part of the model not present in the crystal, showing the interruption of the strand due to strand-swapping. On the right side the 2Fo-Fc electron density shows a continuity of the electron density across the subunits suggesting strand-swapping. (**B**) The same models as in (A) are shown, using the same coloring scheme but stick representation. The non-swapped version on the left where residues ^59^ELS^61^ form a simple contact between adjacent Pbs corresponds to the situation in Ad2, whereas the right panel shows the extensive interaction due to the strand-swapping as observed for Ad3. (**C**) Side-view of the contacts next to a 2-fold symmetry axis of the Dd showing the model of two Pb molecules without swapped N-termini on the left (as in Ad2) and with swapped N-termini on the right (Ad3). (**D**) Inside view of a half Dd (6 Pbs). The surface of the bottom Pb is shown in green, the five interacting Pbs are shown in magenta/violet. The left panel shows the structure for Ad2 without strand-swapping, the right panel shows the 5 N-terminal extensions interacting through strand-swapping.

However, during refinement of the Ad3 model based on the Ad2 Pb structure it became obvious that there was an interruption of the peptide chain between residues 59 to 62, and Fo-Fc and 2Fo-Fc electron density maps suggested an alternative trace (Figure 3AB). This trace corresponds to an exchange of the N-terminal residues 48–61 between monomers related by a 2-fold axis of the dodecahedron (Figure 3C). This strand-swapping, first noticed in the cubic form, was confirmed in the orthorhombic form by generating unbiased maps from lower resolution by 60-fold averaging and by phase extension, starting with model phases that did not include the contact region. Due to methodological limitations it cannot be excluded that non strand-swapped dimers are present in low proportion (<30%) in either crystal form, however the strand swapped form dominates. The strand-swapping was not detected in the cryoEM structure of Ad3 Dd due to the lower resolution, but the cryoEM reconstruction agrees perfectly with the crystallographic model, in particular in the Pb-Pb contact regions and around the trefoil openings (Figure S2). There is however no evidence that this strand-swapping occurs for Ad2 Dd.

This finding implies that instead of just the small contact surface observed for Ad2 Dd, which only forms under very specific crystallization conditions but not under physiological conditions, an extensive interaction involving 27 residues takes place between adjacent Pbs and locks the dodecahedron together (Figure 3CD). For one Pb monomer, the surface area buried in the contact thus increases from 670 (in Ad2 Dd) to 1950 Å^2^ (in Ad3 Dd) and likely explains the formation of stable Ad3 Dd.

### Characterization of Dd assembly mutants

The Pb–Pb interaction regions (Figure 2AB) were used to design mutants, in order to probe the molecular basis of Dd integrity. Firstly, in order to disrupt the interaction of regions 2 and 3, the charge of the involved amino acids was switched in the mutants D100R and R425E. Secondly, as the residue Arg428 is a serine in non-dodecahedron forming Pbs, the mutant R428S has also been studied. Finally, the sequence located at the site of strand-swapping, ^59^ELS^61^, was mutated to ^59^DVA^61^. This triple mutant was designed on the basis of the originally deposited incorrect Ad3 Pb sequence (^59^DVS^61^), with the aim of investigating the effect of a S61A mutation as the sequences of Pb of serotypes which do not form dodecahedra have an alanine at position 61. Nevertheless, the mutation still probes the importance of this region for Dd assembly. Recombinant protein production in the baculovirus system was similar for wild-type Dd and the R425E and R428S mutants, but with an approximately halved production for the ^59^DVA^61^ and D100R mutants. Protein distribution on the sucrose density gradient showed a profile similar to rwtDd for the R428S protein, with much smaller Dd amounts for ^59^DVA^61^, D100R and R425E mutants (see dense sucrose gradient fractions 7–11 in Figure 2C). Ion exchange purification was performed for pooled gradient fractions 7–11. A large amount of ^59^DVA^61^, D100R and R425E proteins was eluted from the column at 280 mM NaCl corresponding to free Pb. The small amount of material recovered for these mutants at 380 mM NaCl corresponding to Dd was concentrated and analyzed. Native gel analysis (Figure 2D) showed that the R428S mutant particles ran with the same mobility as rwtDd, whereas most of the D100R material migrates more slowly and the R425E material was polydisperse with a lower mobility than *bona fide* rwtDd. The ^59^DVA^61^ sample did not show any significant traces of material with normal rwtDd mobility, instead it showed a badly resolved band with the mobility of free penton bases. EM analysis showed only free bases in the ^59^DVA^61^ sample, partly assembled Dd in the D100R sample, a mixture of a small amount of Dd with a large amount of free bases in R425E, and very regular dodecahedra in the R428S sample (Figure 2E). Dd formed with the R428S mutant looks as regular as rwtDd and its proteolysis pattern and cell penetration ability are similar to rwtDd (results not shown). In contrast, D100R Dd is less stable than the wild-type. This might be explained by the fact that the D100R mutant is no longer able to form a salt bridge with Arg428 and an electrostatically favorable interaction is replaced by the repulsion between the two arginines at positions 425 and 100.

**Figure 4:**
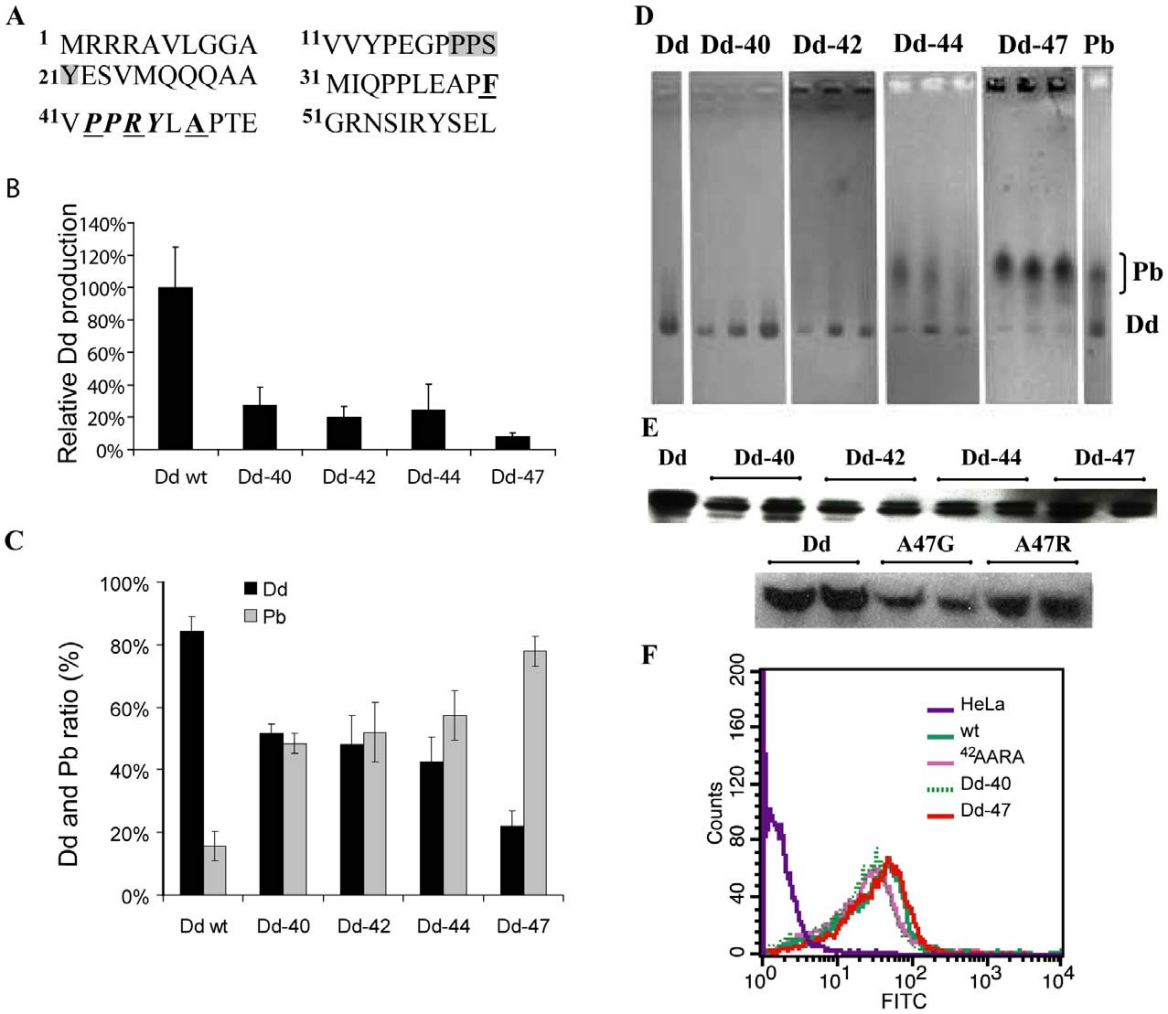
Analysis of Ad3 Pb mutants. (**A**) Sequence of Ad3 Pb N-terminus. The first PPxY motif is underlain in grey, the second PPxY motif is written in bold italics. The first amino acid residue of deletants is written in bold and underlined. (**B**) Dd production in the baculovirus system. Cell lysates were purified by centrifugation on a sucrose density gradient. Fractions recovered from 15–25% sucrose (Pb) and from 30–40% sucrose (Dd) were pooled separately, run on SDS-PAGE and analyzed by gel densitometry. (**C**) Relative production of Dd and Pb by N-terminal deletants. Data for Dd and free Pb production are expressed as a percentage of total Pb protein expression. The average from four electrophoretic runs is shown. (**D**) Native agarose gel electrophoresis (CBB stain) of proteins recovered from the Q-Sepharose column at a salt concentration corresponding to the elution of Dd. The fractions with the highest amount of protein were used. (**E**) Internalization of Dd mutants. Q-Sepharose-purified proteins were applied onto HeLa cells and intracellular Dd was visualized by Western blot or by flow cytometry (**F**) as described in Materials and Methods.

This analysis shows that not all residues presumably implicated in Dd stability have comparable roles. The mutation ^59^DVA^61^ results in the complete lack of Dd formation, while mutations of Arg425 or Asp100 reduce the number of particles. On the other hand, the mutation R428S has no detrimental effect on dodecahedron formation, despite the fact that serine at this position is normally found in non-Dd forming serotypes. Based on sequence alignments of Dd forming and non-forming serotypes, residue Ala47 was also targeted. In the position equivalent to Ala47 an arginine or a glycine is found in the Pbs of Ad serotypes unable to form Dd. However the A47G and A47R Dd mutants did not demonstrate any marked difference from wild-type with respect to Dd formation and cell entry (see Figure 4E).

### Role of the Pb N-terminus in Ad3 dodecahedron formation

Several N-terminal deletion mutants of Ad3 Pb yielding proteins starting with residue 40, 42, 44 or 47 respectively in the vicinity of the second ^42^PPxY motif have have been constructed (Figure 4A). Concomitantly with the length reduction of the N-termini, Dd production was significantly impaired (Figure 4B) although the mutants are still functional as judged from their capacity to be internalized by cells (Figure 4EF). Importantly, in comparison with rwtDd, the ratio of Dd/Pb synthesis strongly diminished for mutants Dd-40>Dd-42>Dd-44≫Dd-47 (Figure 4CD). Mutants that produced higher proportions of Pb have a severely truncated second ^42^PPxY motif of unknown function. We postulated that this motif may play a role in Dd formation or stability through protein-protein interactions with host cell proteins. However, this series of experiments did not allow us to discriminate between the effect of the removal of the PPxY motif and shortening of the Pb N-terminus. Therefore, a Dd mutant was analyzed in which the second PPxY motif, ^42^PPRY^45^, was mutated to the sequence ^42^AARA^45^, thus eliminating this motif without shortening the Pb N-termini. The Dd production of the AARA mutant did not seem to be affected (data not shown), which suggests that decrease of Dd production in the deletion mutants was due to the shortening of Pb N-terminus and not to the elimination of the PPxY motif. As the removal of 47 N-terminal residues prevents Dd assembly (Figure 4BCD), this suggests that several residues before Pro48 must be involved indirectly in strand swapping.

### Localization of the Pb N-terminus

To date, the localization of the 47 N-terminal residues of the Pb within the Dd is not evident from the structural analyses of Ad2 or Ad3 Dd. The first residue visible in the Ad3 Dd atomic structure is Pro48, which is inside the central core of Dd close to the 5-fold axis, suggesting an internal localization of the N-termini. On the other hand, the central volume of 270 000 Å^3^ is too small to accommodate the 60 disordered N-termini. Furthermore, as described above, we used the ^18^PPSY^21^ motif on Dd N-termini to internalize various cargos fused to WW domains, partners of PPxY motifs [6], [9], [19], which suggests an external localization for this part of the N-terminus.

**Figure 5:**
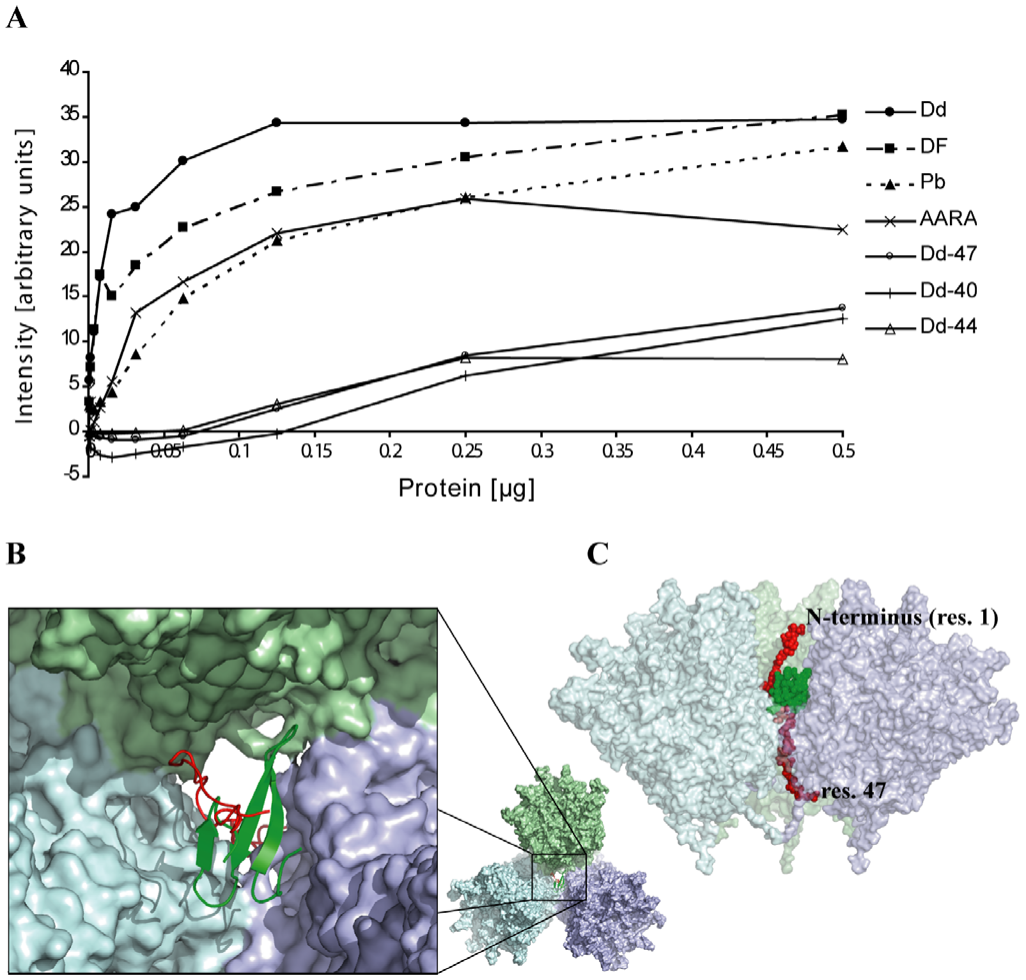
Interaction of Dd with WW protein. (**A**) Aliquots of serially diluted proteins applied in duplicate to a nitrocellulose membrane were overlaid with GST-WW protein as described in Materials and Methods. Interacting GST-WW protein was detected with anti-GST-HRP antibody. The average amount of interacting WW protein was determined by densitometry. (**B**) Cartoon of the trefoil opening between three Pb pentamers shown in pastel colors. A WW domain (pdb 2jo9 [38]) in green is shown bound to the ^19^PPxY motif of the disordered N-terminus (residues 1–47) shown in red. (**C**) The same model viewed from the side represented with a transparent surface. The N-terminus (residues 1–47) is shown in red and the WW domain in green, both as space-filling models. Residues 22–47 can only be seen thanks to the transparent surface of the Pbs. The model does not involve any precise docking but permits visualizing possible positions and relative sizes of the involved molecules.

In order to answer the question of the localization of the Pb N-termini and their potential role in Dd structure stabilization, increasing amounts of recombinant DF and Dd as well as free recombinant pentameric Pb and N-terminal deletants of Dd were incubated in a filter binding assay with the WW domains of yeast Rsp5 protein fused to GST. Binding was quantified with an anti-GST antibody. The best WW domain interactors were DF and Dd, followed by free penton bases and the ^42^AARA^45^ mutant, indicating that the accessibility of at least the first N-terminal ^18^PPxY^21^ motif is required for the interaction with the WW domain (Figure 5A). The N-terminal deletants (Dd-47, Dd-40, Dd-44) that are devoid of the first PPxY motif showed low level binding at higher protein concentrations which was similar to background. This experiment demonstrates that distal regions of the Pb N-termini are accessible for interaction in Dd and suggest that they are located outside the particle's central cavity. Indeed, the Dd structure shows that the N-terminus can extend far enough through the trefoil openings to be accessible to WW domains, as modeled in Figure 5BC.

## Discussion

Dodecahedron-base is a small icosahedral virus-like particle composed of 12 copies of the Ad3 pentameric Pb protein and is capable of efficient cell entry. Our goal was the characterization of structural features responsible for Dd assembly and stability.

Through careful examination of the crystal structure, it was determined that residues 48–61 of Ad3 Pb, the Dd building blocks, interlock within the particle through strand-swapping (Figure 3). This is a key feature distinguishing Ad3 Pb that spontaneously forms Dd from Ad2 Pb, which does not. The strand-swapping considerably increases the contacts between neighboring Pb pentamers which otherwise associates only through three small loop regions (Figure 2AB). Strand-swapping leads to an extensive interaction between residues Pro48 and Ser61 of a polypeptide (Figure S3BD) from one Pb with a two-fold related Pb. This aspect of the Dd interior is reminiscent of some viral capsids, where the exchange of N-terminal arms between capsomers is observed (for example in calicivirus [20]). Likewise, adenovirus trimeric hexons are stabilized by interlocking N-termini [16].

Based initially on the atomic structure of Ad2 dodecahedra formed only under conditions of crystallogenesis [13], we mutated some amino acid residues located in contact regions 2 and 3 between individual Pb pentamers as well as the sequence ^59^ELS^61^ where strand-swapping occurs. These mutations yielded three different phenotypes (Figure 2). Mutant R428S appeared to yield a regular Dd preparation similar to rwtDd, while mutations R425E or D100R induced an attenuated phenotype with purification-unstable Dd, resulting in increased amount of free Pbs in the final preparations. Mutant ^59^DVA^61^ produced only free Pb, indicating the importance of this region for Dd integrity.

The crystallographic analysis helps to explain these results. Residue Arg428, although it is located in region 3, is not directly involved in the hydrogen bonding network between Pbs in the contact region. Its mutation to serine creates an additional putative hydrogen bond with Glu105 on a neighboring pentamer (Figure S3AC). Residues Arg425 and Asp100 are involved in charged interactions and the hydrogen bond network between two pentamers (Figure 2B) that will be disrupted by either mutation, resulting in strongly decreased Dd formation. Finally, the ^59^DVA^61^ mutation is located precisely at the site of strand-swapping. It likely prevents strand-swapping leading to a loss of the stabilizing effect.

As shortening of the N-terminus strongly affects Dd formation, we hypothesize that the fine-tuning of strand-swapping *versus* the absence of strand exchange is dependant on residues 40–47. These residues may be involved, for example, in the formation of folding intermediates, which govern the Ad3 Dd assembly during the formation of the network of sixty interlocked Pb N-termini. The ordered residues 48–65 contain the critical ^59^ELS^61^ motif and add two small β-strands (residues 56–58, β1 and 62–65, β0) to a β-sheet of the jellyroll fold involving strands β2 and β11 (Figure 1A and Figure S3BD). Residues 48–65 are thus not involved in the folding of the jellyroll domain, which starts at residue 66 only. Thus, it is likely that the residues 48–65 can fold into place only when the rest of the protein is already folded.

Despite the very small inter-Pb interface involving regions 2 and 3 compared to the much larger interface generated by strand-swapping, the former contacts are quite important, as demonstrated by the low yield and the instability of the D100R and R425E mutants. This suggests a model of Ad3 Dd assembly where firstly a less stable intermediate is formed through formation of the weak contacts mediated by regions 2 and 3. Subsequently, the N-terminal strand exchange takes place locking the dodecahedral arrangement into an interwoven stable particle. For Ad2 Pb, the first event takes place under crystallization conditions but the N-termini do not rearrange, so that additional agents such as dioxane and ammonium sulphate are needed for Dd stabilization [13]. Interestingly, strand-swapping can occur only in the context of Dd, not in the context of the Ad viral capsid where individual Pb pentamers located at the vertices of the capsid are separated by several hexons. Indeed, the structure of the Ad5 virus particle shows that Pb residues 48 to 61 are not in a strand-swapped conformation [16].

In conclusion, based on our structural and biochemical results, the following elements govern Ad3 Dd stability: (i) the sequence in regions 2 and 3, (ii) the sequence at the site of the strand exchange between residues 58 and 61 and (iii) the length of the disordered N-terminus. The contact residues in region 2 and 3 are compatible with Dd formation for both Ad2 and Ad3, and the absence of Dd formation in Ad2 is unlikely to be explained by the minor serine to alanine change in region 1, the site of strand-swapping. Therefore, the difference between the two Pbs must thus reside principally in the disordered N-terminus, potentially through its involvement in folding intermediates.

As the first residue visible in the Dd crystal structure is Pro48 which is localized inside the main core of the particle this suggested a localization of the N-terminus within the Dd internal cavity. However, not only various cargo proteins can be attached to Dd through an interaction with the first PPxY motif in the Pb N-terminal end (residues 18–21), but also different fusion proteins containing WW domains were efficiently internalized via interaction with the ^18^PPxY motif [6], [9], [18], [19]. Importantly, our WW domain overlay experiment showed that both Dd or Pb that contain an intact ^18^PPxY motif are able to interact with GST-WW protein, while deletants devoid of the motif showed little or no interaction (Figure 5A). The 27 residues between the ^18^PPxY motif and the first visible N-terminal residue (Pro48) can easily span the distance of about 50 Å required to render the motif accessible for the binding of WW domains if the flexible N-terminus passes through the trefoil opening between Pb pentamers. This would also solve the problem of the insufficient volume available inside the particle to accommodate 60 N-termini of 47 residues each. The cavity inside the dodecahedron provides a volume of about 270 000 Å^3^. The total molecular weight of the disordered residues (assuming an absence of proteolysis) equals 310 kDa, in consequence only 0.86 Å^3^Da^−1^ are available to accommodate the termini compared to the average specific volume of 1.23 Å^3^Da^−1^ inside a folded protein [21]. In conclusion, either a part of the N-termini must be located outside the inner cavity or must be proteolyzed in order to allow Dd assembly. Our biochemical and mutagenesis data suggest that the distal part of the N-terminal extension bearing the ^18^PPxY motif, in both recombinant DF and Dd, can be accessible for interaction. Altogether, these data act as a critical guide in the design and manipulation of Ad3 Dd as an *in vivo* delivery vehicle for therapeutic agents.

It has been recently reported that DF binding to Ad3 receptor, desmoglein 2, results in cell remodeling [2]. The Ad3 fiber does interact with this receptor but is unable on its own to trigger cell remodeling [3]. This suggests that multivalent Dd provides the correct spatial arrangement of the fibers, which enables not only interaction with the receptor but also signal transduction necessary for cell remodeling. This seems to be a unique property of dodecahedron-forming adenovirus serotypes. Non Dd-forming serotypes, such as Ad2, use excess fiber protein to disrupt the tight junction through interactions with the cellular receptor CAR [4]. Only Ad3 dodecahedra have been shown to change the distribution of cell surface receptors. This property is of interest for cancer treatment as shown by the enhancement of the therapeutic efficacy of monoclonal antibody treatment when combined with DF or with dimerized Ad3 fiber [22], [23]. Our data suggest that the stabilization of Ad3 Dd by strand-swapping permits Ad3-infected cells to produce excess DF [1], enabling its role in the epithelium disruption [3], likely necessary for virus spread. Based on the studies presented here, a molecular basis of DF assembly and stability is provided which can be directly applied to the design of therapeutic vectors for targeted drug/protein delivery to host cells.

## Methods

### Crystallization and structure determination

Ad3 penton base was produced in insect cells (High-Five) using the baculovirus system and purified over a sucrose density gradient as described previously [5]. Cubic crystals: The protein was crystallized using the hanging drop method (Table S1). Crystals grew over a period of over one year. Crystals were cryo-protected using successive transfers to reservoir solutions with increasing amounts of glycerol (5%, 10%, 15%, 20%), and were frozen in the 100 K nitrogen gas stream. A complete dataset to 4.75 Å was collected on one crystal (Table S1) at the European Synchrotron Radiation Facility (ESRF). Data were processed using MOSFLM [24] and SCALA. The structure was solved with Amore [25] using a C_α_ model of a full dodecahedron of Ad3 obtained by cryo-electron microscopy (pdb entry 2c9g [14]) based on the Ad2 dodecahedron structure (pdb entry 1×9p [13]). The cubic crystals contain 4 pentamers (20 Pb monomers) in the asymmetric unit (asu). A full model of the asu was generated and residues were mutated to the recently corrected Ad3 Pb sequence (GenBank acc. no ABB17799.1 [26]). The model was subjected to rigid body refinement of the individual monomers. Non-crystallographic symmetry (ncs) matrices were derived using LSQKAB [27] in CCP4i [28]. One reference monomer was corrected using COOT [29]. The model was refined at 4.75 Å resolution in CNS1.1 [30] with positional and temperature factor refinement using strict ncs constraints. Electron density maps were calculated using SIGMAA-weighted [31] coefficients obtained from REFMAC [32] after an expansion of the monomer model to a full asu. The electron density was averaged 20 times [33] using an envelope representing the asu of 20 monomers and analyzed around the reference monomer. When strand switching became obvious (Figure 3A) the model was adjusted accordingly.

Orthorhombic crystals: The protein was produced as before [5] and crystallized using the hanging drop method in presence of a 2∶1 molar excess of the peptide corresponding to the Ad2 fiber tail (MKRARPSEDTFNPVYPYDTEC) added to the Ad3 Dd protein prior to crystallization. Crystals grew over a period of 2–4 weeks at 4°C. Crystals were cryo-protected using well solution with 20% sucrose added and flash-frozen in liquid nitrogen. A dataset to 3.8 Å (Table S1) was collected on two crystals at the ESRF. Data were processed using XDS [34] and scaled with XSCALE [35]. The structure was solved using the C_α_ backbone of the 4.75 Å structure of the Ad3 Dd obtained from the cubic crystal form. The orthorhombic crystal form contains a full Dd (60 monomers) per asu. Model correction, refinement and map calculation were done as for the cubic crystal form. A round of REFMAC using 60-fold tight ncs constraints has been used to improve the geometry. Maps were averaged 60-fold within an envelope covering the 60-mer in order to avoid effects at the border of the envelope but maps were analyzed around the reference monomer where ncs operators are the most accurate. Additional electron density in the peptide-binding groove visible in averaged Fo-Fc maps has been modeled using the sequence NPVYPYDTEC of the added peptide. The occupancy was set to 0.8 in order to obtain comparable temperature factors for the atoms of the peptide and neighboring residues of the Pb structure. Figures have been prepared with Pymol (www.pymol.org) and alignments used ESPript [36].

### Dodecahedron production and purification for biochemical assays

Baculoviruses carrying Ad3 Pb wt genes or N-terminally deleted or mutated Ad3 Pb genes were amplified as described [5]. Clarified lysates were fractionated on the sucrose density gradients as previously described [5]. Dd was purified as described [8]. Briefly, gradient fractions between 30–40% sucrose were pooled, dialyzed against 20 mM Tris, pH 7.5, containing 2 mM EDTA, 5% glycerol, Complete protease inhibitors (Roche) and subjected to ion-exchange chromatography on a Q-Sepharose column (High Q Cartridge, Bio-Rad). Proteins eluted with a NaCl gradient were stored at 4°C in dialysis buffer, containing 280 mM NaCl for Pb fractions and 370 mM NaCl for fractions containing Dd. The resulting preparations were thus devoid of cellular DNA which eluted at higher salt concentration than Dd. N-terminal sequencing analysis showed that in all preparations Dd is partially proteolyzed at the N-terminus (see [8]).

### Protein electrophoresis, antibodies and immunological analyses

Fractions recovered from 15–25% sucrose (first 5 fractions) and from 30–40% sucrose (last 5 fractions) were pooled separately and aliquots were analyzed by SDS-PAGE or native agarose electrophoresis [37]. Gels were stained first with ethidium bromide for analysis of DNA contamination and then with Coomassie Brilliant Blue (CBB). For the comparison of Dd and Pb amounts produced by the deletants (Figure 4BC), the dense sucrose gradient fractions were fractionated on a Q-Sepharose column as described above, and the peaks of Pb (low NaCl) and Dd (high NaCl) were resolved by native agarose electrophoresis as shown in Figure 4D and analyzed by densitometry of gels scans (GS 800 Calibrated Densitometer, Biorad).

For Western blot analysis, a polyclonal rabbit anti-Ad3 dodecahedron antibody (Ab) prepared in the laboratory was used at 1∶40 000. A secondary anti-sheep Ab conjugated to horseradish peroxidase (Sigma) was used at 1∶160 000. ECL detection (Amersham Pharmacia Biosciences) was used throughout this work. When needed, intensities of bands were estimated with a densitometer.

### Electron microscopy

Protein samples at approximately 0.1 mg/ml were applied to the clean side of carbon on mica (carbon/mica interface) and negatively stained with 1% sodium silicotungstate, pH 7.0. Micrographs were taken under low-dose conditions with a Jeol 1200 EX II microscope at 100 kV and a nominal magnification of 40 000.

### Dd mutants

Point mutations were introduced into the wt Pb sequence using QuikChange XL Site-Directed Mutagenesis Kit (Stratagene) according to the manufacturer's instructions, with pFastBacDualAd3base plasmid as the PCR template (primers given in Table S3). Ad3 Pb N-terminal deletants were amplified by PCR from wt Pb gene using Pb40, Pb42, Pb44 and Pb47 primers (Table S3) and cloned into pFastBacDual vector. Recombinant baculoviruses were produced using the Bac-to-Bac (Invitrogen) system.

### Cells

HeLa cells were cultured by classical methods under 5% CO_2_ atmosphere in EMEM supplemented with 10% fetal calf serum (FCS), penicillin (50 IU/ml) and streptomycin (50 µg/ml) at 37°C.

### Dd internalization

Portions of purified proteins (1–4 µg/5⋅10^4^cells) were applied on HeLa cells in EMEM without serum. After 90 min incubation at 37°C with rwt or mutant Dd, cells were washed with sterile PBS and detached from culture dish by treatment with 2 mM EDTA in PBS. Cells were further lysed in Laemmli buffer and analyzed by SDS-PAGE followed by Western blot with anti-Dd serum as described above. In parallel, a part of the cells were prepared for flow cytometry. Cells fixed in 100% cold methanol were incubated with the primary anti-Dd antibody (1∶1000 in PBS; 1 h at room temperature), washed with PBS and then incubated with goat anti-rabbit secondary FITC-labeled antibody (Santa Cruz) (1∶200; 1 h at room temperature). After several washes with PBS, aliquots of approximately 10 000 cells were analyzed by flow cytometry on a FACSCalibur (Beckton-Dickinson).

### GST-WW protein purification

GST-WW protein was obtained as described [9]. Briefly, *E. coli* Rosetta 2 competent cells were transformed with pGEX-4-T1 bearing the GST-WW 1,2,3 (Rsp5) gene, cultured to an OD of 0.6, and protein production was induced with 0.4 mM IPTG. After 4 h, cells were harvested by centrifugation and the pellet was treated with BugBuster–Benzonase (Novagen) lysis reagent. The supernatant was applied to a HiTrap GST affinity column (GE Healthcare) connected to a BioLogic (Biorad) FPLC system. Purified GST-WW protein was eluted with 10 mM glutathione in 50 mM Tris, pH 8. The glutathione was subsequently removed by dialysis.

### Analysis of the interaction of the Pb N-terminus with WW domains

rwt DF and Dd, free Pb, N-terminal deletants of Dd and BSA (negative control) were cascade diluted and applied onto a nitrocellulose membrane (Amersham). Dot-blotted proteins were allowed to interact for 2 hours at room temperature with fusion protein GST-WW at 200 µg/ml in 20 mM Tris pH 7.5, 150 mM NaCl, 2 mM EDTA, 5% glycerol. After several washes and blocking with 5% milk in Tris-saline-Tween-20 buffer, the membrane was incubated with anti-GST-HRP antibody (1∶50000; GE Healthcare) for 1 h at 37°C. Visualization of interacting WW protein was performed with ECL system (Sigma), followed by exposure to ECL Hyperfilm (GE Healthcare). Dots were scanned and analyzed using a BioRad Scanner and Quantity One 4.6 software.

## Supporting Information

Table S1 **Data collection statistics.**
(PDF)

Table S2 **Structure refinement and model validation.**
(PDF)

Table S3 **Oligonucleotides used to generate Dd mutants.**
(PDF)

Figure S1 **Structure of Dd with bound fiber peptide.** (**A**) The 60-fold averaged Fo-Fc electron density map (calculated prior to the inclusion of the fiber peptide) is shown together with a cartoon of the Ad3 Pb structure. The modeled peptide (sequence FNPVYPYDTEC) is shown as stick model (orange carbon atoms). (**B**) Superposition of the peptide-bound Pb structures of Ad2 (cyan, pdb entry 1×9t [13] and that of Ad3 (magenta, peptide in orange). (**C**) Positive 60-fold averaged Fo-Fc difference electron density on the 5-fold axis next to Glu451 in the orthorhombic crystal forms contoured at 10 σ. The presence of Ca^2+^ in the buffer suggests a bound Ca^2+^ ion.
(PDF)

Figure S2 **Comparison of the Ad3 Dd cryo-EM** [**14**] **and the X-ray structure (this study).** (**A, B**) iso-surface representation of the 9 Å resolution Ad3 Dd cryo-EM reconstruction (accession number EMDB 1178, pdb 2C9G [14]) viewed respectively down from a 2-fold (A) and a 3-fold (B) axis. In (A), the asterisk highlights the main interaction between two Pbs. In (B) a trefoil opening on the 3-fold axis of the Dd is shown. (**C**) Fit of the Ad3 Pb X-ray structure into the EM density showing the contact between two Pbs (top) and a view down a 5-fold axis from the outside of the particle (bottom, one Pb only). The asterisk highlights the same interaction between two Pbs as in part (A). The arrow points to the region of the strand-swapping between two Pbs that is compatible with the observed electron density.
(PDF)

Figure S3 **The Pb-Pb interface.** (**A**) Modeled interaction of the R428S mutant. The same view and colors as in Figure 2B are used. (**B**) Secondary structure elements involving the N-terminal residues 48 to 65. Colors as in Figure 3, secondary structure elements are labeled as in Figure 1A. (**C**) Stereoview of the same part of the structure as in panel A, the corresponding 60-fold averaged 2Fo-Fc electron density at 3.8 Å resolution is contoured at 1 σ. (**D**) Stereoview of the same part of the structure as in panel B together with electron density as in panel C.
(PDF)

## References

[1] Norrby E (1969) The relationship between the soluble antigens and the virion of adenovirus type 3. IV. Immunological complexity of soluble components. Virology 37: 565–576. 4976534 10.1016/0042-6822(69)90274-8

[2] Wang H, Li ZY, Liu Y, Persson J, Beyer I, et al (2010) Desmoglein 2 is a receptor for adenovirus serotypes 3, 7, 11 and 14. Nat Med 17: 96–104. 21151137 10.1038/nm.2270 PMC3074512

[3] Fender P, Hall K, Schoehn G, Blair GE (2012) The impact of human adenovirus type 3 dodecahedron on host cells and its potential role in viral infection. J Virol 86: 5380–5385. 22345476 10.1128/JVI.07127-11 PMC3347364

[4] Walters RW, Freimuth P, Moninger TO, Ganske I, Zabner J, et al (2002) Adenovirus fiber disrupts CAR-mediated intercellular adhesion allowing virus escape. Cell 110: 789–799. 12297051 10.1016/s0092-8674(02)00912-1

[5] Fender P, Ruigrok RW, Gout E, Buffet S, Chroboczek J (1997) Adenovirus dodecahedron, a new vector for human gene transfer. Nat Biotechnol 15: 52–56. 9035106 10.1038/nbt0197-52

[6] Garcel A, Gout E, Timmins J, Chroboczek J, Fender P (2006) Protein transduction into human cells by adenovirus dodecahedron using WW domains as universal adaptors. J Gene Med 8: 524–531. 16389639 10.1002/jgm.862

[7] Fender P, Schoehn G, Foucaud-Gamen J, Gout E, Garcel A, et al (2003) Adenovirus dodecahedron allows large multimeric protein transduction in human cells. J Virol 77: 4960–4964. 12663801 10.1128/JVI.77.8.4960-4964.2003 PMC152161

[8] Zochowska M, Paca A, Schoehn G, Andrieu JP, Chroboczek J, et al (2009) Adenovirus dodecahedron, as a drug delivery vector. PLoS One 4: e5569. 19440379 10.1371/journal.pone.0005569 PMC2679213

[9] Naskalska A, Szolajska E, Chaperot L, Angel J, Plumas J, et al (2009) Influenza recombinant vaccine: matrix protein M1 on the platform of the adenovirus dodecahedron. Vaccine 27: 7385–7393. 19766576 10.1016/j.vaccine.2009.09.021

[10] Ishizu KI, Watanabe H, Han SI, Kanesashi SN, Hoque M, et al (2001) Roles of disulfide linkage and calcium ion-mediated interactions in assembly and disassembly of virus-like particles composed of simian virus 40 VP1 capsid protein. J Virol 75: 61–72. 11119574 10.1128/JVI.75.1.61-72.2001 PMC113898

[11] Chen PL, Wang M, Ou WC, Lii CK, Chen LS, et al (2001) Disulfide bonds stabilize JC virus capsid-like structure by protecting calcium ions from chelation. FEBS Lett 500: 109–113. 11445066 10.1016/s0014-5793(01)02598-4

[12] McCarthy MP, White WI, Palmer-Hill F, Koenig S, Suzich JA (1998) Quantitative disassembly and reassembly of human papillomavirus type 11 virus-like particles in vitro. J Virol 72: 32–41. 9420197 10.1128/jvi.72.1.32-41.1998 PMC109346

[13] Zubieta C, Schoehn G, Chroboczek J, Cusack S (2005) The structure of the human adenovirus 2 penton. Mol Cell 17: 121–135. 15629723 10.1016/j.molcel.2004.11.041

[14] Fuschiotti P, Schoehn G, Fender P, Fabry CM, Hewat EA, et al (2006) Structure of the dodecahedral penton particle from human adenovirus type 3. J Mol Biol 356: 510–520. 16375921 10.1016/j.jmb.2005.11.048

[15] Fender P, Boussaid A, Mezin P, Chroboczek J (2005) Synthesis, cellular localization, and quantification of penton-dodecahedron in serotype 3 adenovirus-infected cells. Virology 340: 167–173. 16040074 10.1016/j.virol.2005.06.030

[16] Liu H, Jin L, Koh SB, Atanasov I, Schein S, et al (2010) Atomic structure of human adenovirus by cryo-EM reveals interactions among protein networks. Science 329: 1038–1043. 20798312 10.1126/science.1187433 PMC3412078

[17] Galinier R, Gout E, Lortat-Jacob H, Wood J, Chroboczek J (2002) Adenovirus protein involved in virus internalization recruits ubiquitin-protein ligases. Biochemistry 41: 14299–14305. 12450395 10.1021/bi020125b

[18] Gout E, Gutkowska M, Takayama S, Reed JC, Chroboczek J (2010) Co-chaperone BAG3 and adenovirus penton base protein partnership. J Cell Biochem 111: 699–708. 20607728 10.1002/jcb.22756 PMC7166384

[19] Villegas-Mendez A, Garin MI, Pineda-Molina E, Veratti E, Bueren JA, et al (2010) In vivo delivery of antigens by adenovirus dodecahedron induces cellular and humoral immune responses to elicit antitumor immunity. Mol Ther 18: 1046–1053. 20179681 10.1038/mt.2010.16 PMC2890114

[20] Chen R, Neill JD, Estes MK, Prasad BV (2006) X-ray structure of a native calicivirus: structural insights into antigenic diversity and host specificity. Proc Natl Acad Sci U S A 103: 8048–8053. 16702551 10.1073/pnas.0600421103 PMC1472427

[21] Matthews BW (1968) Solvent content of protein crystals. J Mol Biol 33: 491–497. 5700707 10.1016/0022-2836(68)90205-2

[22] Wang H, Li Z, Yumul R, Lara S, Hemminki A, et al (2011) Multimerization of adenovirus serotype 3 fiber knob domains is required for efficient binding of virus to desmoglein 2 and subsequent opening of epithelial junctions. J Virol 85: 6390–6402. 21525338 10.1128/JVI.00514-11 PMC3112237

[23] Beyer I, van Rensburg R, Strauss R, Li Z, Wang H, et al (2011) Epithelial junction opener JO-1 improves monoclonal antibody therapy of cancer. Cancer Res 71: 7080–7090. 21990319 10.1158/0008-5472.CAN-11-2009 PMC3217128

[24] Leslie AG (1999) Integration of macromolecular diffraction data. Acta Crystallogr D Biol Crystallogr 55: 1696–1702. 10531519 10.1107/s090744499900846x

[25] Navaza J, Saludjian P (1997) AMoRe: An automated molecular replacement program package. In: C W Carter J, Sweet RM, editors. Methods in Enzymology. London & New York: Academic Press. pp. 581–594. 10.1016/S0076-6879(97)76079-8 27799116

[26] Sirena D, Ruzsics Z, Schaffner W, Greber UF, Hemmi S (2005) The nucleotide sequence and a first generation gene transfer vector of species B human adenovirus serotype 3. Virology 343: 283–298. 16169033 10.1016/j.virol.2005.08.024 PMC7172737

[27] Kabsch W (1976) A solution for the best rotation to relate two sets of vectors. Acta Cryst A 32: 922–923.

[28] Potterton E, Briggs P, Turkenburg M, Dodson E (2003) A graphical user interface to the CCP4 program suite. Acta Crystallogr D Biol Crystallogr 59: 1131–1137. 12832755 10.1107/s0907444903008126

[29] Emsley P, Cowtan K (2004) Coot: model-building tools for molecular graphics. Acta Crystallogr D Biol Crystallogr 60: 2126–2132. 15572765 10.1107/S0907444904019158

[30] Brunger AT, Adams PD, Clore GM, DeLano WL, Gros P, et al (1998) Crystallography & NMR system: A new software suite for macromolecular structure determination. Acta Crystallogr D Biol Crystallogr 54 (Pt 5) 905–921. 9757107 10.1107/s0907444998003254

[31] Read RJ (1986) Improved Fourier coefficients for maps using phases from partial structures with errors. Acta Crystallographica Section D Biological Crystallography A 42: 140–149.

[32] Murshudov GN, Vagin AA, Lebedev A, Wilson KS, Dodson EJ (1999) Efficient anisotropic refinement of macromolecular structures using FFT. Acta Crystallographica Section D Biological Crystallography 55: 247–255. 10089417 10.1107/S090744499801405X

[33] Stein PE, Boodhoo A, Armstrong GD, Cockle SA, Klein MH, et al (1994) The crystal structure of pertussis toxin. Structure 2: 45–57. 8075982 10.1016/s0969-2126(00)00007-1

[34] Kabsch W (2010) Xds. Acta Crystallogr D Biol Crystallogr 66: 125–132. 20124692 10.1107/S0907444909047337 PMC2815665

[35] Kabsch W (2010) Integration, scaling, space-group assignment and post-refinement. Acta Crystallogr D Biol Crystallogr 66: 133–144. 20124693 10.1107/S0907444909047374 PMC2815666

[36] Gouet P, Courcelle E, Stuart DI, Metoz F (1999) ESPript: analysis of multiple sequence alignments in PostScript. Bioinformatics 15: 305–308. 10320398 10.1093/bioinformatics/15.4.305

[37] Gallegos CO, Patton JT (1989) Characterization of rotavirus replication intermediates: a model for the assembly of single-shelled particles. Virology 172: 616–627. 2552662 10.1016/0042-6822(89)90204-3

[38] Yamasaki M, Li W, Johnson DJ, Huntington JA (2008) Crystal structure of a stable dimer reveals the molecular basis of serpin polymerization. Nature 455: 1255–1258. 18923394 10.1038/nature07394

