# Supplementary material for: The Structural Basis for the Integrity of Adenovirus Ad3 Dodecahedron
Source: PLoS One. 2012 Sep 25;7(9):e46075. doi: 10.1371/journal.pone.0046075 (PMC3457955; doi:10.1371/journal.pone.0046075)
Supplement: Table S1 — Data collection statistics. (PDF) [file pone.0046075.s001.pdf]

Table S1. Data collection statistics. Values for the outer shell are given in parentheses.

	**Cubic crystals**<br><br>Spacegroup $P2_13$	**Orthorhombic crystals**<br><br>Spacegroup $P2_12_12_1$
Crystallization	Reservoir 12 % PEG 8000, 100 mM HEPES pH 6.5, 100 mM $CaCl_2$	Reservoir 10-15 % PEG 8000, 100 mM HEPES pH 6.5, 100 mM $CaCl_2$
Cell parameters (Å)	a=342.7 b=342.7 c=342.7	a=346.0 b=348.6 c= 372.9
X-ray source	ESRF beamline ID14-3	ESRF beamline ID23-1
Wavelength (Å)	0.931	1.033
Detector	MarCCD 133	MarCCD 225
Temperature (K)	100.0	100
Resolution range (Å)	69.95 - 4.75 (5.01–4.75)	30.0 – 3.8 (3.9 – 3.8)
No. of unique reflections	67599 (9808)	249444 (5040)
Completeness (%)	100 (100)	56.8 (42.1)
Redundancy	5.7 (5.7)	2.36 (1.29)
$< I/\sigma(I)>$	3.3 (1.3)	4.67 (0.91)
R_{merge}	0.212 (0.582)	0.173 (0.714)
