# Supplementary material for: The Structural Basis for the Integrity of Adenovirus Ad3 Dodecahedron
Source: PLoS One. 2012 Sep 25;7(9):e46075. doi: 10.1371/journal.pone.0046075 (PMC3457955; doi:10.1371/journal.pone.0046075)
Supplement: Table S2 — Structure refinement and model validation. (PDF) [file pone.0046075.s002.pdf]

Table S2. Structure refinement and model validation. Values for the outer shell are given in parentheses.

	Cubic crystals	*Orthorhombic crystals*
pdb entry	4aqq	4ar2
Resolution range (Å)	69.95 - 4.75 (4.87 - 4.75)	20.0 – 3.8 (3.9 – 3.8)
No. of reflections used in refinement	67517 (4489)	244404 (12572)
Final overall *R* factor	0.291	0.280
Atomic displacement model	isotropic	isotropic
Overall average *B* factor ($Å^2$)	56.4	71.8
Rmsd bonds (Å)	0.016	0.015
Rmsd angles (°)	2.5	2.4
Final R_{work}	0.291 (0.328)	0.280 (0.454)
No. of reflections for R_{free}	3418 (267)	5040 (266)
Final R_{free}	0.290 (0.321)	0.278 (0.452)
