# Supplementary material for: The Structural Basis for the Integrity of Adenovirus Ad3 Dodecahedron
Source: PLoS One. 2012 Sep 25;7(9):e46075. doi: 10.1371/journal.pone.0046075 (PMC3457955; doi:10.1371/journal.pone.0046075)
Supplement: Table S3 — Oligonucleotides used to generate Dd mutants. (PDF) [file pone.0046075.s003.pdf]

Table S3. **Oligonucleotides used to generate Dd mutants.** Changed codons are in bold. All mutations and constructs were confirmed by DNA sequence analysis.

**Mutation**	**Direction**	**Sequence (5' – 3')**
A38G	Forward	CCCCACTGGAG**GGT**CCCTTCGTACC
A38G	Reverse	GGTACGAAGGG**ACC**CTCCAGTGGGG
A38P	Forward	AGCCCCCACTGGAG**CCT**CCCTTCGTACC
A38P	Reverse	GGTACGAAGGG**AGG**CTCCAGTGGGGGCT
A47R	Forward	CACGGTACCT**GCG**TCCTACGGAAGG
A47R	Reverse	CCTTCCGTAGGA**CGC**AGGTACCGTG
A47G	Forward	CACGGTACCT**GGG**TCCTACGGAAGG
A47G	Reverse	CCTTCCGTAGGA**CCC**AGGTACCGTG
^{59}DVA^{61}	Forward	CATTCGTTACTCG**GACGTGGCT**CCCCTGTACGATACCACC
^{59}DVA^{61}	Reverse	GGTGGTATCGTACAGGGG**AGCCACGTC**CGAGTAACGAATG
D100R	Forward	CGGTGGTGCAGAACAAT**CGT**TTTACCCCCACGGAGGC
D100R	Reverse	GCCTCCGTGGGGGTAAA**ACG**ATTGTTCTGCACCACCG
R425E	Forward	CAAGACCCAGTCACCTTC**GAG**TCCACAAGACAAGTCAAC
R425E	Reverse	GTTGACTTGTCTTGTGGA**CTC**GAAGGTGACTGGGTCTTG
R428S	Forward	CACCTTCCGCTCCACA**TCC**CAAGTCAACAACTACC
R428S	Reverse	GGTAGTTGTTGACTTG**GGA**TGTGGAGCGGAAGGTG
Pb-9	Forward	GAGAATTCATGGGAGCGGTGGTG
Pb-9	Reverse	GTTTCAGGTTCAGGGGGAGG
Pb-40	Forward	CGGAATTCTATGTTCGTACCCCCACGGT
Pb-40	Reverse	GTTTCAGGTTCAGGGGGAGG
Pb-42	Forward	TAGAATTCATGCCCCCACGGTACCTGGC
Pb-42	Reverse	GTTTCAGGTTCAGGGGGAGG
Pb-44	Forward	GAGAATTCCATGCGGTACCTGGCGCCTA
Pb-44	Reverse	GTTTCAGGTTCAGGGGGAGG
Pb-47	Forward	TCGAATTCCCACGGTACATGGCGCCTAC
Pb-47	Reverse	GTTTCAGGTTCAGGGGGAGG
^{42}AARA^{45}	Forward	GCTCCCTTCGTA**GCCGCT**CGG**GCC**CTGGCGCCTACG
^{42}AARA^{45}	Reverse	CGTAGGCGCCAG**GGCCCG**AGC**GGC**TACGAAGGGAGC
