# Supplementary figures and images for: The Structural Basis for the Integrity of Adenovirus Ad3 Dodecahedron
Source: PLoS One. 2012 Sep 25;7(9):e46075. doi: 10.1371/journal.pone.0046075 (PMC3457955; doi:10.1371/journal.pone.0046075)

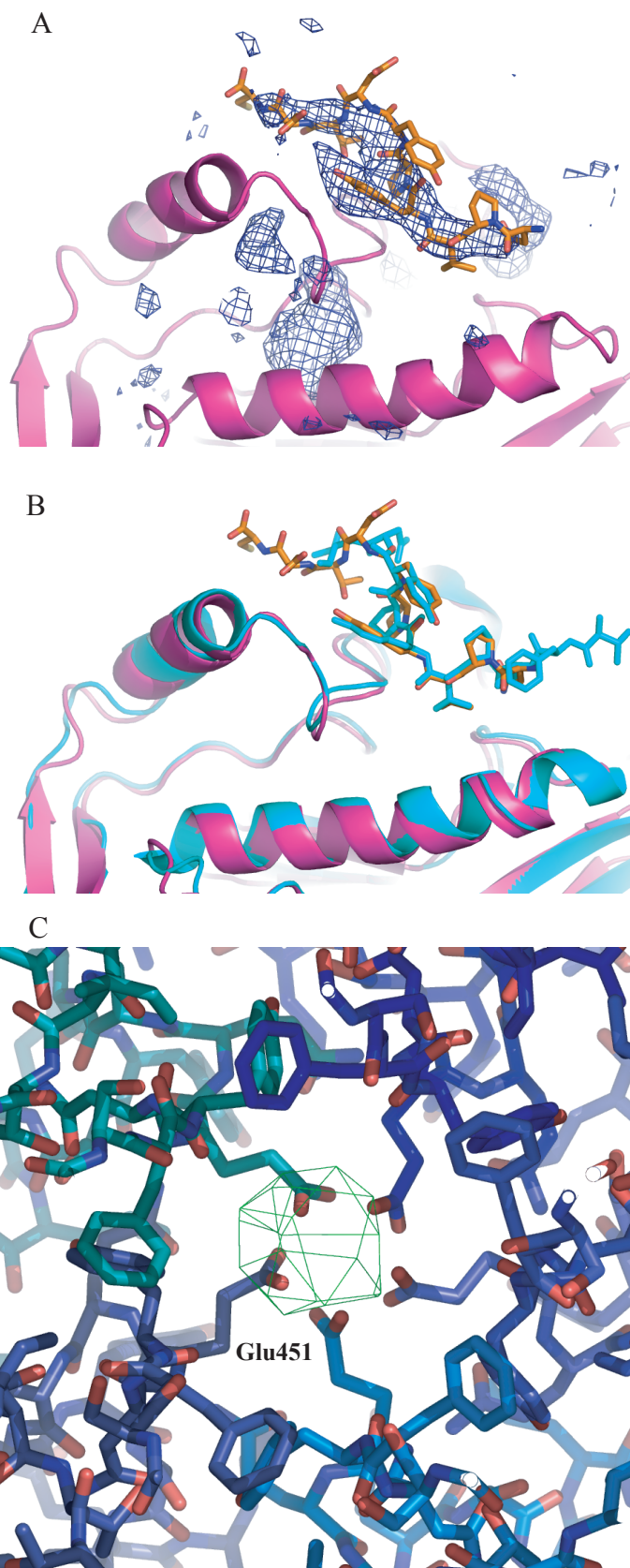

Figure S1

Supplement: Figure S1 — Structure of Dd with bound fiber peptide. (A) The 60-fold averaged Fo-Fc electron density map (calculated prior to the inclusion of the fiber peptide) is shown together with a cartoon of the Ad3 Pb structure. The modeled peptide (sequence FNPVYPYDTEC) is shown as stick model (orange carbon atoms). (B) Superposition of the peptide-bound Pb structures of Ad2 (cyan, pdb entry 1×9t [13] and that of Ad3 (magenta, peptide in orange). (C) Positive 60-fold averaged Fo-Fc difference electron density on the 5-fold axis next to Glu451 in the orthorhombic crystal forms contoured at 10 σ. The presence of Ca2+ in the buffer suggests a bound Ca2+ ion. (PDF) [file pone.0046075.s004.pdf]

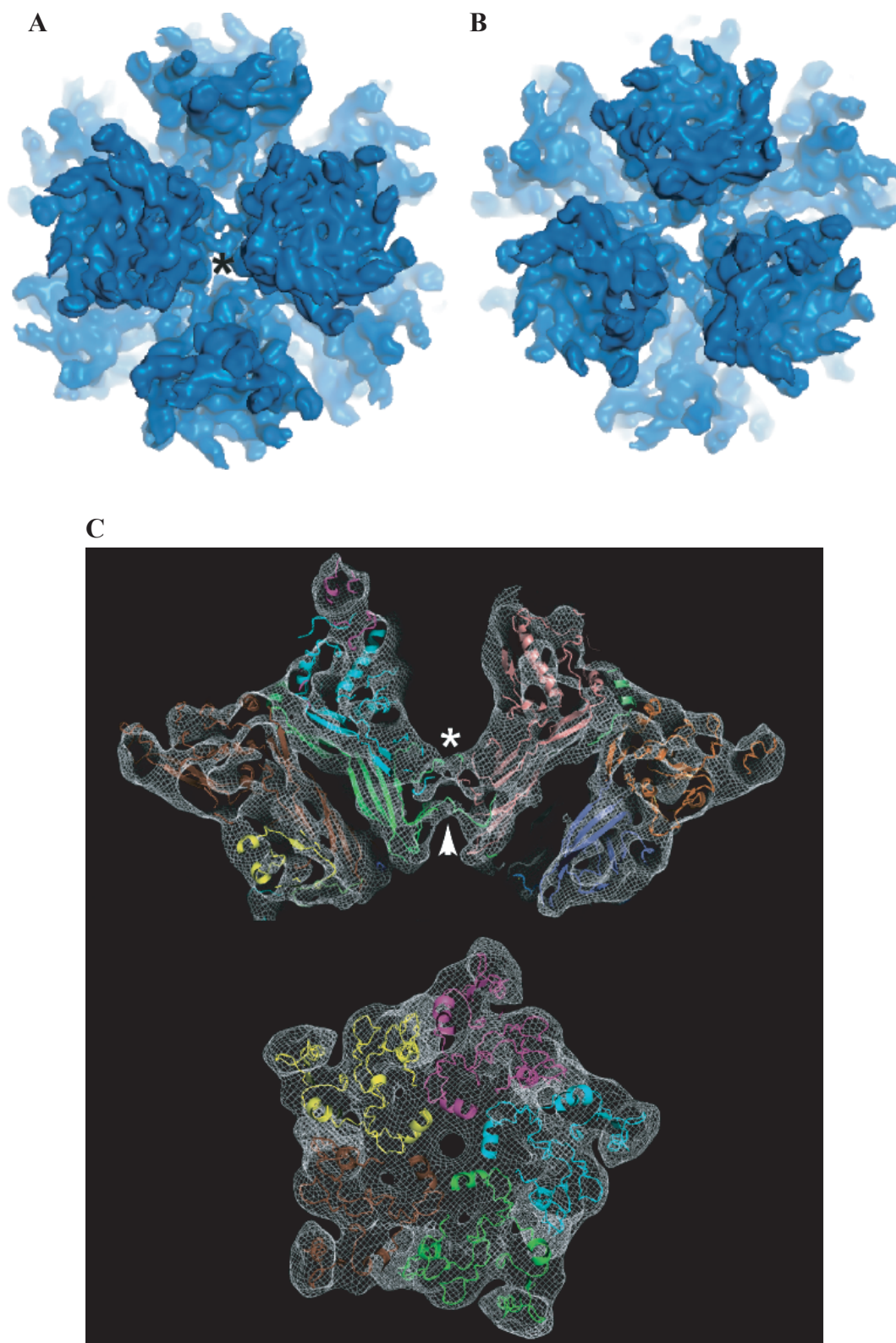

Figure S2

Supplement: Figure S2 — Comparison of the Ad3 Dd cryo-EM [14] and the X-ray structure (this study). (A, B) iso-surface representation of the 9 Å resolution Ad3 Dd cryo-EM reconstruction (accession number EMDB 1178, pdb 2C9G [14]) viewed respectively down from a 2-fold (A) and a 3-fold (B) axis. In (A), the asterisk highlights the main interaction between two Pbs. In (B) a trefoil opening on the 3-fold axis of the Dd is shown. (C) Fit of the Ad3 Pb X-ray structure into the EM density showing the contact between two Pbs (top) and a view down a 5-fold axis from the outside of the particle (bottom, one Pb only). The asterisk highlights the same interaction between two Pbs as in part (A). The arrow points to the region of the strand-swapping between two Pbs that is compatible with the observed electron density. (PDF) [file pone.0046075.s005.pdf]

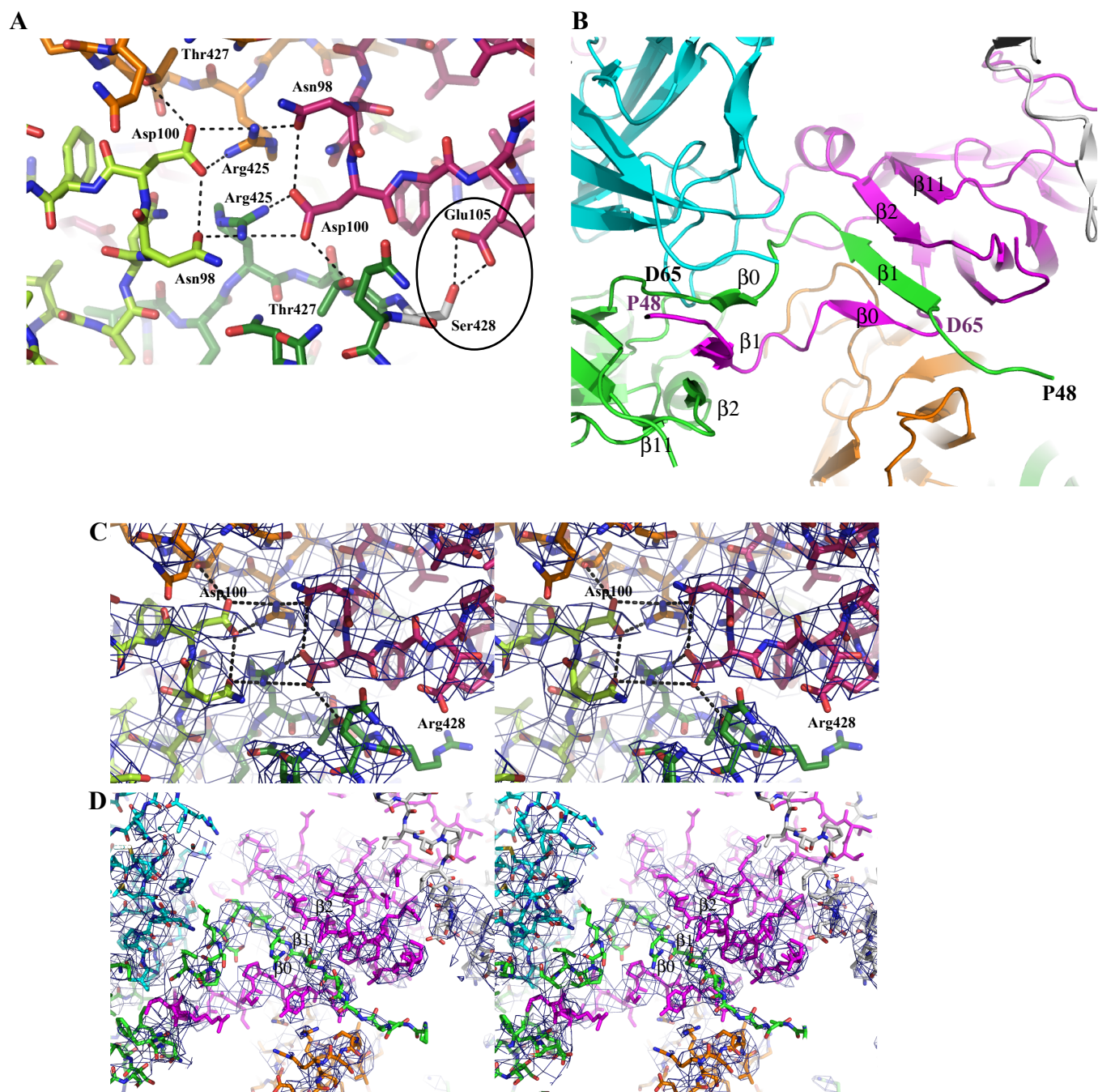

Figure S3

Supplement: Figure S3 — The Pb-Pb interface. (A) Modeled interaction of the R428S mutant. The same view and colors as in Figure 2B are used. (B) Secondary structure elements involving the N-terminal residues 48 to 65. Colors as in Figure 3, secondary structure elements are labeled as in Figure 1A. (C) Stereoview of the same part of the structure as in panel A, the corresponding 60-fold averaged 2Fo-Fc electron density at 3.8 Å resolution is contoured at 1 σ. (D) Stereoview of the same part of the structure as in panel B together with electron density as in panel C. (PDF) [file pone.0046075.s006.pdf]
